# The Association of Cytomegalovirus IgM and Allostatic Load

**DOI:** 10.3390/diseases10040070

**Published:** 2022-09-27

**Authors:** Matthew Hill, Emmanuel Obeng-Gyasi

**Affiliations:** 1 Department of Built Environment, North Carolina A&T State University, Greensboro, NC 27411, USA; 2 Environmental Health and Disease Laboratory, North Carolina A&T State University, Greensboro, NC 27411, USA

**Keywords:** allostatic load, cytomegalovirus, stress, CMV IgM, infection

## Abstract

Background: Cytomegalovirus (CMV) is a deoxyribonucleic acid virus that affects a significant proportion of the worldwide population; after primary infection, it goes into a latent state and can be reactivated, primarily after a reduction in host immune defenses. Methods: This study evaluated the association of acute cytomegalovirus infection (CMV IgM) and Allostatic Load (AL) by sociodemographic factors using data from the National Health and Nutrition Examination Survey (NHANES) 2001–2004 among participants (aged 20–49 years). CMV infection was determined by the level of CMV IgM antibody in serum samples. AL was assessed as a combination of 10 biomarkers from the cardiovascular, inflammatory, and metabolic systems. The evaluation of the association between CMV infection and AL included descriptive statistics and logistic regression models, which were adjusted for demographic and behavioral covariates. Results: AL was more elevated among those who were older, male, those with lower education, those performing limited physical activity, and smokers. CMV was more elevated in females than males among those who consumed alcohol and cigarette smokers. In Pearson’s correlation analysis, there was a slight positive correlation between CMV IgM and AL, with triglycerides and Body Mass Index (BMI) the most strongly correlated with AL. Binary logistic regression showed no significant relationship between high AL and positive CMV IgM but did show a significant relationship between high AL and age (OR = 1.0592, 95% CI 1.0215–1.0983, *p* = 0.00715). The findings of this study provide insight into the relationship between CMV and AL and provide awareness of factors that affect their relationship.

## 1. Introduction

### 1.1. Epidemiology of Cytomegalovirus

Cytomegalovirus (CMV) is a highly prevalent virus in the herpesvirus family, which affects nearly half of the world’s population [1]. It also infects roughly half of the US population and can be spread through bodily fluids such as blood, breast milk, or saliva. CMV has no known cure, although it is mostly harmless to healthy individuals. Once infected, a person retains a latent infection for the remainder of their life. This infection can rotate through periods of dormancy, and it may reactivate when factors such as immunosuppression and inflammation occur [2]. To best measure past infection, the CMV seropositivity of an individual is used [3]. Race may be a critical factor in CMV exposure risk. Previous studies have shown that the seroprevalence of CMV tends to be about 20–30% higher in non-whites versus whites [1]. Age-adjusted CMV seroprevalence rates in the US are about 81% for Mexican Americans and 75% for African Americans. CMV is widespread, yet the two groups most at risk are those with compromised immune systems and pregnant women, as it infects the placenta, crosses the placental barrier, and adversely affects the embryo and developing fetus, potentially resulting in adverse pregnancy outcomes [4]. This can lead to hearing loss, vision loss, and mental dysfunction. Exposed individuals with poorer health status are prone to negative effects such as increased rates of infections and elevated all-cause mortality. One study among intensive care unit (ICU) patients found that those with CMV infection had 1.93 times the odds for all-cause mortality [5].

#### Clinical/Laboratory Diagnosis

CMV presents with non-specific clinical symptoms; thus, laboratory examination is the primary basis for diagnosing CMV infection. CMV virus replication and serum immunological methods are the two most widely used methods [6]. CMV-specific immunoglobulin M (IgM) is a sensitive and specific indicator of an ongoing or recent CMV infection [7,8,9]

### 1.2. Allostatic Load and Health

Allostatic load (AL), an index of chronic stress, is the culmination of stress on the body and the physiological adaptations [10,11]. Operationalization of AL is often study-specific and generally involves summing biomarkers from the neuroendocrine, metabolic, cardiovascular, and immune systems into an AL Index [12].

The concept of AL is related to allostasis and homeostasis. Allostasis posits that individuals adjust to the demands of the environment [13] and setpoints of physiological normality fail to capture the dynamic environments and physiological responses individuals must operate within. On the other hand, homeostasis defines health as a state in which all physiological parameters must operate within non-changing setpoints. For example, homeostasis would posit that blood pressure higher than the normality setpoint of 120/80 mmHg is elevated, while one observing the concept of allostasis would speculate that set points are not critical and an individual who is, for example, sprinting, should have a higher blood pressure than, i.e., 120/80 mmHg because the demands of the moment are higher. With allostasis, individuals respond appropriately to challenges. However, if the challenges are continuous and do not turn off, the body adapts at the higher set point. Regarding stress, when the setpoint changes, it is called AL. AL can compromise the immune system, which may allow latent infections to reappear or enable individuals to be infected [14].

### 1.3. Sociodemographic Factors and Allostatic Load

The AL of a person can also be determined by certain sociodemographic factors, such as income, gender, and race/ethnicity [15,16]. For example, an individual with lower socioeconomic status may, in turn, have poor living conditions, which can lead to unhealthy behaviors, i.e., smoking and drinking [15]. In addition, an individual’s race has been shown to be a factor in AL in US adults [16,17]. In fact, Black adults tend to have higher AL compared to White and Mexican Americans [16]. This difference can be mainly attributed to early health deterioration associated with repeated sociodemographic adversity and political marginalization, commonly referred to as the weathering hypothesis [17,18].

### 1.4. Sociodemographic Factors and CMV Exposure Risk

CMV exposure risk has been shown to be correlated with an individual’s age and the female sex [19]. This disadvantage is possibly due to hormonal changes older females tend to undergo as opposed to males. Females are also at a disadvantage when it comes to CMV seroconversion, the changing from states of seronegative to seropositive [19]. For example, seronegative females also show a nearly three-fold difference in ages 12–19 as compared to other age groups [20].

An individual’s race is also important to consider when examining CMV exposure risk [21]. In fact, the US non-Hispanic Blacks and Mexican Americans have been found to have a CMV rate of infection greater than five as opposed to non-Hispanic Whites with a 1.4 risk factor [20]. Black individuals have also been shown to have a much higher frequency of CMV infection than white individuals [21]. Some of these differences can be linked to health disparities between racial groups [22]. An additional factor linked with CMV exposure risk is education [23]. Lower household education has been shown to be linked with CMV seroprevalence [23,24]. Those with the lowest education level had a CMV seroprevalence of 49.3% as opposed to 37.5% for individuals with a college/university diploma/degree [23].

Another important concept to consider is how a person’s CMV exposure risk may be impacted by their country of origin [25]. For example, in the Eastern Mediterranean region, CMV seroprevalence is highest at about 90%, and in the Western Pacific and African regions, CMV seroprevalence is about the same at 88% [1].

The financial condition of an individual has a significant impact on their access to healthcare as well as their living conditions, and people with lower incomes tend to have higher CMV prevalence worldwide [22]. These serve as additional stressors that impact the immune system of an individual.

### 1.5. Study Objectives

In this study, we hypothesized that sociodemographic factors are critical variables in the relationship between CMV and AL. The objective of this study was to use data obtained from the National Health and Nutrition Examination Survey (NHANES) 2001–2004 to firstly (a) explore the relationship between AL and Sociodemographic factors such as age, race/ethnicity, education, and the ratio of family income to poverty, next, we (b) examined the relationship between CMV and the aforementioned sociodemographic factors. Finally, we (c) examined the relationship between AL and CMV, considering the effects of these factors. This study is critical as little is known about these relationships among US Adults. With a mean seroprevalence of 83% for the general population, a better understanding of CMV and its interaction with stress (AL) by sociodemographic factors is crucial [26].

## 2. Materials and Methods

### 2.1. Population in Study

The study sample was obtained from NHANES 2001–2004. Our study had inclusion criteria of individuals (aged 20–49 years) who were tested for CMV and had all markers within the AL Index. NHANES 2001–2004 uses a complex, multistage, stratified, and clustered sampling approach to survey non-institutionalized US persons. For this study, the total number of sampled participants was 2595, of which 662 were used due to data availability.

### 2.2. Operationalizing Allostatic Load

To measure this stress, we use an AL index consisting of the following biomarkers, systolic blood pressure (SBP), diastolic blood pressure (DBP), total cholesterol (TC), high-density lipoprotein (HDL) cholesterol, glycosylated hemoglobin (HbA1c), as well as albumin (Alb), triglyceride (TG), body mass index (BMI), creatinine clearance (CLCR), and C-reactive protein (CRP). AL markers were divided into quartiles based on their distribution within the database. High risk for each biomarker was the top 25% in the distribution for all markers apart from albumin, creatinine clearance, and HDL cholesterol, for which the bottom 25% of the distribution was considered to have the highest risk. Every eligible individual with the required data in the study was assigned a value of 1 if they were in the high-risk category or a 0 if in the low-risk category for all markers to calculate a total AL value out of 10. Clinical and laboratory collection and analysis of markers and variables of interest have been described elsewhere [27]. An AL value greater than or equal to 3 was considered high per the literature [28,29].

### 2.3. Measurement of Study Variables

The demographic data were gathered via the NHANES computer-assisted personal interview (CAPI) software program. The data included gender, age, race/ethnicity, income, and education.

To measure CMV lgM, trained personnel used an ELISA assay by Diamedix, Miami Lakes, FL, and the automated analyzer MAGO. Confirmatory testing for CMV IgM was performed on specimens within a wide range above and below the MAGO test cutoff using the VITEK Immunodiagnostic Assay System (VIDAS) ELISA assay by bioMerieux, Inc., Durham, NC. Creatinine was measured using Jaffé rate reaction on the CX3 analyzer. Urine albumin was measured using a solid-phase fluorescent immunoassay. A1C was measured using the Primus instrument, a fully automated glycohemoglobin analyzer, which utilizes the principle of boronate affinity high-performance liquid chromatography (HPLC) (Primus Corporation). CRP was measured by latex-enhanced nephelometry. Fasting total serum cholesterol and triglycerides were measured enzymatically in serum or plasma in a series of coupled reactions. HDL cholesterol was measured using a heparin-manganese (Mn) precipitation method. Biochemistry biomarkers were measured using a mercury sphygmomanometer.

### 2.4. Description of Study Variables

In this study, variables of interest include CMV lgM, AL, age, race/ethnicity (non-Hispanic (NH) white, NH black, Mexican American, and Other Hispanic, and Other/Multiracial), gender (Male/Female), education, annual family income, alcohol consumption, smoked cigarettes, currently smoking, muscle strengthening activities (physical activity) and the number of times performed. The annual family poverty income ratio was measured as either greater than or equal to, or less than 5. An individual’s education (was represented by the following, less than 9th grade, 9–11th grade (Includes 12th grade with no diploma), High school graduate/General Educational Development (GED) or equivalent, some college or AA degree, college graduate or above, refused, and don’t know. Alcohol consumption was measured as yes if an individual consumed at least 12 alcoholic drinks in the past year and no otherwise. The smoked cigarettes variable measured whether an individual smoked at least 100 cigarettes in their lifetime. Finally, the variable currently smoking was measured as yes for all individuals that had at least one cigarette in the past month.

### 2.5. Statistical Analysis

Statistical analysis for this study was performed using R programming language R (version 4.1.1; R Foundation for Statistical Computing, Vienna, Austria) and accounted for the sampling design and weights of the NHANES dataset. We began by first exploring the summary statistics of these variables to gain an insight into their distributions. The Shapiro–Wilk test was used to test for the normality of the data and determine if any log transformation was necessary. To measure the relationship between variables, logistic regression models were used along with chi-square test of independence. These methods were selected due to the binary and categorical variables present in the NHANES dataset. A *p*-value < 0.05 was considered significant in all our analyses.

## 3. Results

### 3.1. Characteristics of Study Participants

This study explored AL by CMV and sociodemographic factors. Table 1 provides descriptive summaries of the total number of individuals and survey-weighted percentages for categorical variables, as well as means and standard errors in parenthesis for continuous variables by high and low AL. The total number of sample participants was 2595, of which 662 had the data necessary to compute AL risk. All summary statistics are shown using post-stratified population numbers. According to the 2000 census, over 124 million Americans were between the ages of 20 and 49 [30]. Of these individuals, 47.71% were males, and 52.29% were females. The majority of individuals were negative for CMV IgM (98.59 %). The average age of participants was 35.10 years; individuals with low AL were slightly younger (32.99), while those with high AL were older (37.77). The sample consisted of 16.83% Mexican American, no other Hispanics, 60.54% non-Hispanic white, 20.87% non-Hispanic black, and 1.76% of other races.

Critical variables of interest by CMV IgM are shown in Table 2. The results indicated that those positive for CMV IgM tend to be younger, aged 30.4, compared to 35.36 for negative individuals. More women tend to be positive for CMV IgM, and Mexican Americans and other races are most impacted. We also see that, on average, both CMV IgM negative and positive groups tend to have more individuals with low AL.

### 3.2. Association of Allostatic Load and CMV

**Figure 1:**
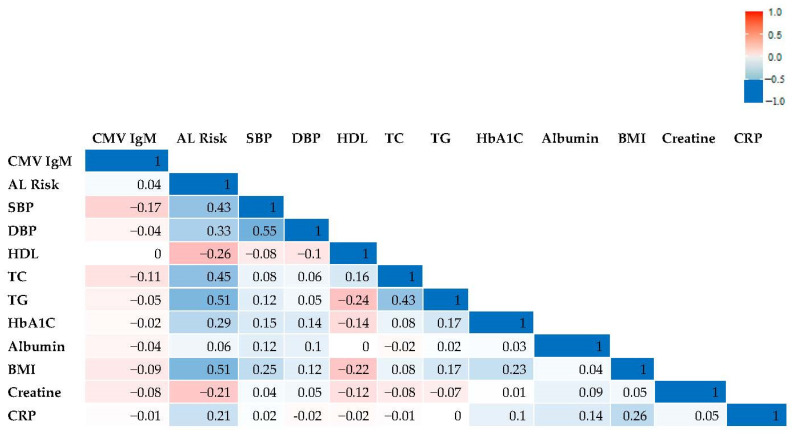
Correlation matrix of AL, CMV, and AL biomarkers (Pearson correlation coefficient).

A correlation matrix was developed to explore the correlation between the critical variables in this study (Figure 1). We performed the correlational analysis to see which variables in AL may be driving associations. The strongest correlation was found between Systolic BP and Diastolic BP. Triglyceride (TG) and BMI were the most strongly correlated with AL (0.51).

Binary logistic regression models were developed to study the association between AL (high/low) and CMV IgM (Table 3). The model was adjusted for age, gender, race/ethnicity, smoking, and Alcohol consumption. The association between AL and CMV IgM was not significant. The model indicated that age was significantly associated with AL.

## 4. Discussion

CMV is a cell-mediated immune function marker and may be linked to stress. AL captures cumulative physiological stress using multiple biomarkers and, by capturing the effects of stressors on several biological systems simultaneously, more reliably models the effects of stress on health outcomes such as cardiovascular disease, physical decline, metabolic dysfunction, and all-cause mortality. Understanding the distribution and determinants of CMV exposure and its association with AL is critical to study in order to improve the health of individuals and populations.

In our study, the proportion for CMV IgM was not significantly different by AL category (high/low AL), possibly indicating that those with high and low AL are being acutely exposed to cytomegalovirus. This also potentially implies the degree to which human CMV is ubiquitous in the US population [31]. CMV though relatively innocuous in immunocompetent adults has been demonstrated to cause complications such as end-organ disease and death in immunocompromised individuals [32]. Nonetheless, in persons with normal immunity, CMV infection has associated with profound stimulation of immune and inflammatory pathways. The degree to which this occurs and its effects on AL may not be significant per our results among adults aged 20–49. This matches the work of Galiatsatos and colleagues, who found that CMV in young, healthy patients had a good prognosis with no intervention [33]. Research by Seeman and colleagues also found age differences in the manifestation of AL [34], which may indicate that our results reflect the age of those within the study rather than the absolute association over the life course.

Indeed, those with high AL tended to be older, and those positive for CMV tended to be younger in our study. AL has been found to increase sharply by age from 20 to 60 [35]. Regarding CMV, studies have observed that the seroprevalence for CMV increases gradually with age, but those sampled in these studies tended to have a larger range of age groups, while our study only looked at those between the ages of 20 through 49 years [36].

Those with high AL were more likely to be male, with females being a larger proportion of CMV IgM-positive individuals. Studies have consistently found AL to be higher in males than females [37,38], with CMV found to either be similar across genders or higher for females [39].

Education was a factor in AL and CMV exposure. Those with low education (9–11th grade with no diploma) made up a significantly larger proportion of those with high AL. For CMV IgM, those negative for it made up a larger proportion of individuals in this education category. For those who indicated their education included some college or an associate degree, those with low AL were the majority in this category. For CMV IgM, those who were positive made up a significant proportion of those who said their education included some college or an associate degree.

In the literature, low education levels have been previously observed as risk factors for increased susceptibility to CMV infection, perhaps through direct contact with contagious secretions from a poor hygiene environment [40,41]. However, these studies examined CMV-specific immunoglobulin G antibodies, while ours examined CMV-specific immunoglobulin M antibodies. This may indicate that acute exposure to CMV differentially affects sociodemographic outcomes compared to chronic exposure. Regarding AL, studies have found that schooling does not itself protect against AL [42]. That said, educational attainment has been found to have a protective effect on AL in later life [43].

There were no significant differences in cigarette smokers comparing high AL vs. low AL. However, those who were CMV IgM positive made up a slightly larger proportion of those who smoked.

CMV IgM positive also made up a large proportion of those consuming significant amounts of alcohol.

Lifestyle coping behaviors such as alcohol and cigarette consumption have been found to significantly contribute to AL [44]. Our results are consistent with Hawkley and colleagues, who found in their sample of adults that smoking (either current or past) was not significantly correlated with AL [45].

Nevertheless, other studies by Gustaffson and colleagues have shown that in adult men, health behaviors, including smoking, attenuate the link between socioeconomic status and AL [46]. Regarding Cytomegalovirus Seropositivity, smoking has been found to be associated with airflow restriction in a cohort of military veterans with an elevated prevalence of smoking [47].

In our study, those not doing significant physical activities made up a larger proportion of high AL, while this did not hold for CMV IgM positive. Physical activity has been noted to be a resilient behavior that lowers AL [10,48]. This may indicate the need to increase physical activity as a mechanism to lessen the effects of AL regardless of the source of exposure.

A slight positive correlation existed between CMV IgM and AL in correlational analysis (Pearson’s correlation). Triglycerides and BMI were the most strongly correlated with AL. This may speak to the need to monitor triglyceride and BMI levels as part of an intervention to decrease AL. Adults with obesity in the US, compared with those with normal weight, experienced higher annual medical care costs [49]; thus, a comprehensive action plan to control AL could have a significant impact on the overall health costs within the US.

Binary logistic regression showed no significant relationship between AL (High/Low) and positive CMV IgM but did show a significant relationship between AL and age (*p* = 0.00715). This is likely due to the stressors that come with aging, which include chronic illnesses, psychosocial stress, cognitive impairment, and finances [50].

### Limitations

This was a cross-sectional study; as such, data were collected in a single period and were not longitudinally collected. The cross-sectional design of the data collection limits the degree to which we can attribute causality, especially regarding temporality. In addition, variables such as sex and age must be further explored to assess these relationships fully. Finally, this study was an analysis of secondary data. Finally, the years chosen for this study were based on data availability, and the available data were collected nearly 20 years ago. This means the current situation may have changed due to environmental and social factors which have occurred over the last two decades.

## 5. Conclusions

This study did not find an association between CMV IgM and AL in US adults aged 20–49 using NHANES data. What was apparent is that sociodemographic factors such as age, gender, education, and behavioral variables such as alcohol consumption and smoking are linked to both AL and CMV IgM, with age being the most significant. Those with high AL were more likely to be older, speaking to the need to tailor stress reduction programs or interventions toward more at-risk adults in the 20–49 age range. Future work should seek to explore these findings in a prospective longitudinal study and assess exposure in a more nuanced way factoring in variables such as timing, duration, and severity of exposure to best understand the impact of AL on CMV acutely. Finally, subsequent studies must look at the effect of age by examining the effects in older middle-aged and older adults, as this study focused on those aged 20–49 who generally tend to be in better health and for whom acute exposure to CMV and AL may manifest differently as compared to an older cohort.

Future work must also critically make progress towards better explaining how sex, occupation, and other workplace and sociodemographic factors may act as a driver of exposure differences and how this can play a role in health disparities. In conclusion, we have provided an important preliminary analysis of the association between AL and CMV, which must serve as a driver for further studies to better understand the relationship between the critical variables in this study.

## Figures and Tables

**Table 1:** Means (standard error) of critical variables by AL (High/Low).

Characteristic	Overall	Low AL (N = 8,100,520)	High AL (N = 6,361,368)
Age	35.10 (8.74)	32.99 (8.33)	37.77 (8.53)
Sex			
Female	7,562,431.22 (52.29%)	4,424,015.71 (54.61%)	3,138,415.51 (49.34%)
Male	6,899,456.71 (47.71%)	3,676,503.88 (45.39%)	3,222,952.82 (50.66%)
Race/Ethnicity			
NH_White	8,755,868.44 (60.54%)	4,713,956.77 (58.19%)	4,041,911.67 (63.54%)
Mexican American	2,433,690.81 (16.83%)	1,508,167.40 (18.62%)	925,523.41 (14.55%)
NH_Black	3,017,833.87 (20.87%)	1,712,032.44 (21.13%)	1,305,801.43 (20.53%)
Other and/or Multiracial	254,494.82 (1.76%)	166,362.99 (2.05%)	88,131.83 (1.39%)
Other Hispanic	0.00 (0.00%)	0.00 (0.00%)	0.00 (0.00%)
Annual Family Income			
USD0–USD4999	798,096.32 (5.67%)	487,910.01 (6.14%)	310,186.31 (5.07%)
USD10,000–USD14,999	1,100,662.64 (7.82%)	728,671.23 (9.17%)	371,991.41 (6.08%)
USD15,000–USD19,999	963,166.68 (6.84%)	579,145.22 (7.29%)	384,021.46 (6.27%)
USD20,000–USD24,999	1,387,409.11 (9.86%)	776,977.53 (9.78%)	610,431.58 (9.97%)
USD25,000–USD34,999	1,442,052.01 (10.25%)	850,959.31 (10.71%)	591,092.70 (9.65%)
USD35,000–USD44,999	1,186,327.32 (8.43%)	646,896.07 (8.14%)	539,431.25 (8.81%)
USD45,000–USD54,999	1,194,416.14 (8.49%)	703,122.98 (8.85%)	491,293.16 (8.02%)
USD5000–USD9999	646,050.03 (4.59%)	323,317.27 (4.07%)	322,732.77 (5.27%)
USD55,000–USD64,999	1,065,592.88 (7.57%)	458,237.05 (5.77%)	607,355.83 (9.92%)
USD65,000–USD74,999	1,033,293.71 (7.34%)	522,564.04 (6.57%)	510,729.67 (8.34%)
USD75,000 and over	3,029,142.64 (21.53%)	1,720,581.87 (21.65%)	1,308,560.77 (21.37%)
Over USD20,000	103,235.58 (0.73%)	59,917.36 (0.75%)	43,318.22 (0.71%)
Under USD20,000	122,412.88 (0.87%)	90,252.31 (1.14%)	32,160.57 (0.53%)
Education Level			
9–11th grade (Includes 12th grade with no diploma)	2,220,959.16 (15.41%)	950,641.84 (11.80%)	1,270,317.32 (19.97%)
College graduate or above	1,934,563.35 (13.42%)	896,940.76 (11.14%)	1,037,622.60 (16.31%)
High school graduate/GED or equivalent	4,695,534.31 (32.57%)	2,835,243.24 (35.20%)	1,860,291.07 (29.24%)
Less than 9th grade	1,122,346.49 (7.79%)	577,767.99 (7.17%)	544,578.50 (8.56%)
Some college or AA degree	4,442,683.58 (30.82%)	2,794,124.73 (34.69%)	1,648,558.85 (25.92%)
Alcohol Consumption			
Yes	10,073,294.98 (73.00%)	5,837,655.26 (75.90%)	4,235,639.72 (69.35%)
No	3,725,709.73 (27.00%)	1,853,574.58 (24.10%)	1,872,135.14 (30.65%)
Currently Smoking			
Yes	4,451,670.38 (66.48%)	2,430,938.28 (68.23%)	2,020,732.10 (64.49%)
No	2,244,241.49 (33.52%)	1,131,684.89 (31.77%)	1,112,556.60 (35.51%)
Cigarette Smoker			
Yes	6,695,911.87 (46.30%)	3,562,623.17 (43.98%)	3,133,288.70 (49.25%)
No	7,765,976.06 (53.70%)	4,537,896.43 (56.02%)	3,228,079.63 (50.75%)
Physical Activity			
No	10,042,801.29 (69.44%)	5,204,263.64 (64.25%)	4,838,537.65 (76.06%)
Unable to Do	153,778.94 (1.06%)	58,332.04 (0.72%)	95,446.90 (1.50%)
Yes	4,265,307.71 (29.49%)	2,837,923.92 (35.03%)	1,427,383.79 (22.44%)
Number of muscle-strengthening activities in the last 30 days	15.45 (18.36)	15.53 (12.10)	15.27 (26.79)
Unknown	10,196,580	5,262,596	4,933,985
CMV IGM			
Negative	14,258,317.17 (98.59%)	7,987,151.56 (98.60%)	6,271,165.61 (98.58%)
Positive	203,570.77 (1.41%)	113,368.04 (1.40%)	90,202.72 (1.42%)
Avg Systolic BP	115.93 (14.14)	111.20 (10.89)	121.96 (15.46)
Avg Diastolic BP	72.03 (11.23)	68.85 (10.15)	76.08 (11.25)
HDL	49.63 (14.44)	53.08 (12.98)	45.23 (15.03)
Total Cholesterol	193.61 (40.78)	181.01 (31.40)	209.66 (45.50)
Triglyceride	132.81 (105.18)	96.51 (48.18)	179.04 (135.68)
HbA1C	5.32 (0.63)	5.17 (0.33)	5.51 (0.83)
Albumin, Urine	34.81 (7.72)	29.06 (10.81)	42.12 (10.32)
BMI	27.84 (6.12)	25.39 (4.49)	30.96 (6.49)
Creatinine, Urine	154.38 (86.36)	168.63 (85.53)	136.25 (84.12)
C-Reactive Protein	0.38 (0.78)	0.26 (0.88)	0.53 (0.60)

**Table 2:** Means (standard error) of critical variables by acute CMV exposure status (IgM+/IgM−).

Characteristic	Overall (N = 58,254,118)	CMV IgM Negative, N = 57,352,826	CMV IgM Positive, N = 901,292
Age	35.29 (8.59)	35.36 (8.57)	30.40 (8.57)
Sex			
Female	32,640,056.76 (56.03%)	31,954,897.51 (55.72%)	685,159.24 (76.02%)
Male	25,614,061.41 (43.97%)	25,397,928.36 (44.28%)	216,133.05 (23.98%)
Race/Ethnicity			
NH_White	35,119,201.45 (60.29%)	34,460,451.74 (60.09%)	658,749.71 (73.09%)
Mexican American	10,285,217.38 (17.66%)	10,188,226.98 (17.76%)	96,990.40 (10.76%)
NH_Black	12,077,990.97 (20.73%)	11,932,438.79 (20.81%)	145,552.18 (16.15%)
Other and/or Multiracial	771,708.36 (1.32%)	771,708.36 (1.35%)	0.00 (0.00%)
Other Hispanic	0.00 (0.00%)	0.00 (0.00%)	0.00 (0.00%)
Annual Family Income			
USD0–USD4999	2,996,142.19 (5.26%)	2,944,929.53 (5.25%)	51,212.66 (5.68%)
USD5000–USD9999	3,367,630.93 (5.91%)	3,271,869.09 (5.83%)	95,761.83 (10.62%)
USD10,000–USD14,999	5,224,259.19 (9.17%)	5,192,909.53 (9.26%)	31,349.65 (3.48%)
USD15,000–USD19,999	4,190,027.73 (7.35%)	4,155,385.69 (7.41%)	34,642.04 (3.84%)
USD20,000–USD24,999	5,183,282.06 (9.09%)	5,098,891.05 (9.09%)	84,391.01 (9.36%)
USD25,000–USD34,999	6,605,132.96 (11.59%)	6,489,300.22 (11.57%)	115,832.73 (12.85%)
USD35,000–USD44,999	5,146,442.16 (9.03%)	5,012,278.56 (8.94%)	134,163.60 (14.89%)
USD45,000–USD54,999	5,390,253.42 (9.46%)	5,332,968.88 (9.51%)	57,284.54 (6.36%)
USD55,000–USD64,999	3,726,513.41 (6.54%)	3,726,513.41 (6.64%)	0.00 (0.00%)
USD65,000–USD74,999	3,395,690.40 (5.96%)	3,351,282.11 (5.97%)	44,408.28 (4.93%)
USD75,000 and over	10,440,089.54 (18.32%)	10,187,843.59 (18.16%)	252,245.94 (27.99%)
Over USD20,000	659,204.30 (1.16%)	659,204.30 (1.18%)	0.00 (0.00%)
Under USD20,000	667,756.64 (1.17%)	667,756.64 (1.19%)	0.00 (0.00%)
Education Level			
9–11th grade (Includes 12th grade with no diploma)	9,471,651.40 (16.27%)	9,421,651.27 (16.44%)	50,000.13 (5.55%)
College graduate or above	9,508,020.97 (16.33%)	9,342,110.06 (16.30%)	165,910.91 (18.41%)
High school graduate/GED or equivalent	16,888,929.50 (29.01%)	16,625,057.36 (29.01%)	263,872.15 (29.28%)
Less than 9th grade	4,082,106.67 (7.01%)	4,068,153.41 (7.10%)	13,953.27 (1.55%)
Some college or AA degree	18,257,608.58 (31.37%)	17,850,052.74 (31.15%)	407,555.84 (45.22%)
Alcohol Consumption			
Yes	20,322,664.55 (73.52%)	19,983,797.49 (73.37%)	338,867.06 (83.29%)
No	7,320,995.64 (26.48%)	7,253,009.44 (26.63%)	67,986.20 (16.71%)
Currently Smoking			
Yes	19,040,473.81 (67.43%)	18,704,233.91 (67.38%)	336,239.90 (70.49%)
No	9,195,523.88 (32.57%)	9,054,753.15 (32.62%)	140,770.73 (29.51%)
Cigarette Smoker			
Yes	28,235,997.68 (48.48%)	27,758,987.05 (48.41%)	477,010.63 (52.93%)
No	29,995,710.66 (51.51%)	29,571,429.00 (51.58%)	424,281.66 (47.07%)
Physical Activity			
No	40,948,234.07 (70.29%)	40,395,511.41 (70.43%)	552,722.66 (61.33%)
Unable to Do	713,335.11 (1.22%)	693,718.06 (1.21%)	19,617.05 (2.18%)
Yes	16,592,548.98 (28.48%)	16,263,596.40 (28.36%)	328,952.58 (36.50%)
Number of times spent muscle strengthening in the last 30 days	14.58 (17.41)	14.45 (17.22)	20.62 (24.71)
Unknown	41,661,569	41,089,229	572,340
Avg Systolic BP	116.52 (14.32)	116.63 (14.36)	109.55 (8.34)
Avg Diastolic BP	71.55 (11.86)	71.59 (11.92)	69.11 (6.34)
HDL	50.21 (14.49)	50.15 (14.49)	54.63 (14.40)
Total Cholesterol	197.03 (41.43)	197.12 (41.43)	191.20 (41.79)
Triglyceride	135.15 (136.51)	135.32 (137.39)	124.23 (58.04)
HbA1C	5.35 (0.75)	5.35 (0.75)	5.37 (1.07)
Albumin, Urine	24.68 (3.10)	24.96 (3.13)	6.54 (0.82)
BMI	28.25 (6.50)	28.28 (6.51)	26.42 (5.86)
Creatinine, Urine	145.30 (86.51)	145.79 (86.73)	113.60 (64.53)
C-Reactive Protein	0.43 (0.85)	0.43 (0.86)	0.40 (0.51)
AL			
Low	8,100,519.60 (56.01%)	7,987,151.56 (56.02%)	113,368.04 (55.69%)
High	6,361,368.33 (43.99%)	6,271,165.61 (43.98%)	90,202.72 (44.31%)

**Table 3:** Results of binary logistic regression for AL on CMV (IgM+/IgM−)—adjusting for the demographic and behavioral factors.

Model Variables	* Adjusted OR (95% CI)	*p*-Value
* CMV IgM		
Positive	0.4241 (0.0445, 4.0445)	0.39834
Negative	Reference	
Age	1.0592 (1.0215 1.0983)	0.00715
Sex		
Male	1.5026 (0.6962, 3.2432)	0.25092
Female	Reference	
Race		
NH White	Reference	
NH Black	0.5427 (0.2532, 1.1631)	0.0998
Mexican American	1.0778 (0.4936, 2.3535)	0.82695
Other/Multiracial	1.9799 (0.0786 49.8863)	0.63203
Alcohol		
Yes	0.7796 (0.2495, 2.4363)	0.62134
No	Reference	
Smoking Now		
Yes	0.9742 (0.3986, 2.3809)	0.94679
No	Reference	

* Adjusted for age, gender, race/ethnicity, smoking, and alcohol consumption.

## Data Availability

The NHANES dataset is publicly available online, accessible at cdc.gov/nchs/nhanes/index.htm (accessed on 12 August 2022).
